# An Improved Tiered Head Pose Estimation Network with Self-Adjust Loss Function

**DOI:** 10.3390/e24070974

**Published:** 2022-07-14

**Authors:** Xiaoliang Zhu, Qiaolai Yang, Liang Zhao, Zhicheng Dai, Zili He, Wenting Rong, Junyi Sun, Gendong Liu

**Affiliations:** National Engineering Research Center of Educational Big Data, Central China Normal University, Wuhan 430079, China; zhuxl@ccnu.edu.cn (X.Z.); yql2020113547@mails.ccnu.edu.cn (Q.Y.); dzc@ccnu.edu.cn (Z.D.); hzlzero@mails.ccnu.edu.cn (Z.H.); rwt_0706@mails.ccnu.edu.cn (W.R.); sunjunyi@mails.ccnu.edu.cn (J.S.); gendong@mails.ccnu.edu.cn (G.L.)

**Keywords:** head pose estimation, angle estimation discontinuity, tiered estimation, loss limitation

## Abstract

As an important task in computer vision, head pose estimation has been widely applied in both academia and industry. However, there remains two challenges in the field of head pose estimation: (1) even given the same task (e.g., tiredness detection), the existing algorithms usually consider the estimation of the three angles (i.e., roll, yaw, and pitch) as separate facets, which disregard their interplay as well as differences and thus share the same parameters for all layers; and (2) the discontinuity in angle estimation definitely reduces the accuracy. To solve these two problems, a THESL-Net (tiered head pose estimation with self-adjust loss network) model is proposed in this study. Specifically, first, an idea of stepped estimation using distinct network layers is proposed, gaining a greater freedom during angle estimation. Furthermore, the reasons for the discontinuity in angle estimation are revealed, including not only labeling the dataset with quaternions or Euler angles, but also the loss function that simply adds the classification and regression losses. Subsequently, a self-adjustment constraint on the loss function is applied, making the angle estimation more consistent. Finally, to examine the influence of different angle ranges on the proposed model, experiments are conducted on three popular public benchmark datasets, BIWI, AFLW2000, and UPNA, demonstrating that the proposed model outperforms the state-of-the-art approaches.

## 1. Introduction

As an important task in computer vision, head pose estimation has been applied in a wide range of applications, such as tiredness detection and autonomous driving. The primary approaches mainly rely on either landmark detection [1,2,3,4,5] or depth information [6,7,8,9]. For example, when building fine 3D face models, the landmark detection approach usually attains 3D and 2D mapping and matching. When depth information is used, the detection approach usually makes up for the missing spatial information in 2D images. The corresponding approaches reveal good robustness despite small-area occlusion, but perform badly when the masking area is extended or there is a large deflection of facial angles [6,10,11,12]. It has also been revealed that by introducing convolutional neural networks (CNNs) into head pose estimation tasks, performance degradation can be enhanced due to missing facial key points [10,11,12,13,14,15,16,17,18]. Among the corresponding approaches, the difficulty is generally addressed by direct regression [16,17,18]. Inspired by the idea of soft stagewise regression in age estimation tasks [19], CNN is applied in head pose estimation tasks with leapfrogging findings [10,11,12,13,14,15].

**Figure 1:**
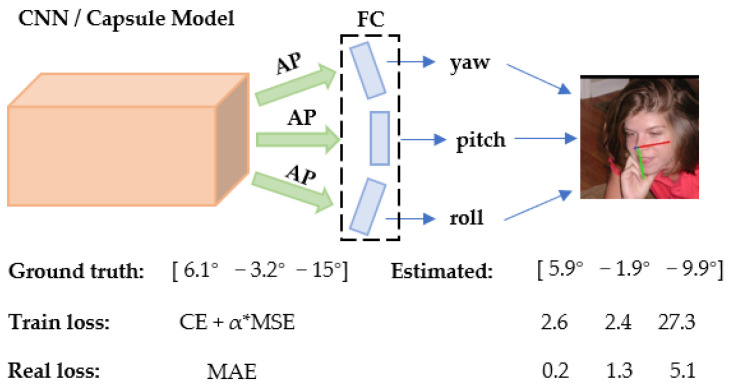
Example of frequent models and loss functions for training and testing on the 300W-LP dataset (AP is the average pooling operation, and FC is a fully connected layer; α equals 1 or 2).

Furthermore, capsule networks [20] are also employed in head pose estimation tasks, which have commonalities with CNN-based works. Among these studies, a balance between the yaw, pitch, and roll is preserved by linearly combining the features extracted from the network, estimating all the three angles simultaneously [6,10,11,12,13,14,15]. The effectiveness of these approaches has been confirmed on numerous public benchmark datasets [21,22,23,24]. Since the parameters of each layer are shared in the three-angle estimation process, although the computational cost is reduced, it leads to the problem of tuning interference between the layers [25,26,27,28,29]. Additionally, recent studies have demonstrated that the use of quaternion or Euler angles (taken as 3D rotational representation labels of datasets) has several shortcomings, leading to discontinuity problems in angle estimation [11,30,31,32]. Meanwhile, the inconsistency in the training and testing loss function is also a cause of discontinuity [11]. To summarize, the reasons for the discontinuity in angle estimation are revealed in Figure 1. Notably, as one of the main causes of the angle estimation discontinuity problem, Euler angles and quaternion labeling are adequately tackled using vector matrices [11,12]. However, neither the tuning interference difficulties between the three angles nor the conflict between loss functions is solved.

For clarification, the two difficulties mentioned above are illustrated. Figure 1 reveals the estimation for a single image from the 300W-LP dataset. The yaw with less expected loss may become worse when the model’s parameters are adjusted using the loss feedback from other angles. When the head pose’s true angles are [6.1°, −3.2°, −15°] and the expected angles are [5.9°, −1.9°, −9.9°], the traditional loss inaccurately reverses the true loss relationship between yaw and pitch, leading to an imbalance of losses on both sides of the classification line. The intermittent nature of the losses and the erroneous inversions make the model tedious to learn; consequently, this problem is discussed and solved further in Section 3 without using the rotation matrix or soft stagewise regression.

In other ways, it has been reported that an imbalance in the dataset’s distribution can damage the model’s performance [33,34,35,36,37,38,39]. To eliminate the imbalance, the oversampling mentioned in [33] is employed, the effect of angle distribution is examined on the BIWI dataset, and then it is compared with datasets that have different angular ranges [24].

Apart from these challenges, some exciting findings have been reached in the studies related to neural networks. Among them, multi-scale feature fusion, as a combination of feature pyramid network [40,41] and feature weight assignment based on attention mechanism [42], has a positive effect on almost all computer vision (CV) tasks [43,44,45]. Additionally, some studies have attempted to enhance the performance of the optimizer [46] and activation function [47], with positive findings. Based on the above studies, a series of advancements are made in this study, aiming to minimize the estimation loss of head pose estimation. In summary, the primary contributions of our study are as follows:

(1) An idea of tiered estimation by combining multi-output task and multi-scale estimation fusion is proposed, which can not only provide greater freedom of adjustment for the three head attitude angles, but also efficiently minimize the interaction between tuning angles and further lower the estimation loss of each angle.
(2) To remove the inconsistency in loss function, which is the main cause of angle estimation discontinuity problems, an easy-to-use dynamic self-adjusting loss function is developed.
(3) To examine the influence of the range of angle distributions on the proposed model, a test is conducted on three public benchmark datasets, demonstrating that our approach maintains remarkable performance for various angle ranges.

The rest of the paper is organized as follows. The existing work on head pose estimation is presented in Section 2. The tiered estimation module and loss limitation method are described in Section 3. The experimental findings on various datasets are depicted in Section 4. Finally, a summary is given in Section 5.

## 2. Related Work

### 2.1. Estimation with Key Points

By matching key facial points, which are recognized from images with 3D face landmarks, the head pose can be computed by the landmark-based approaches. For instance, in [5], every landmark was considered as a separate part, and a tree-structured model was employed to capture the global elastic deformation of the face. In addition, the direct predictive estimation of face landmark positions using an ensemble of regression trees was suggested in [1], which can optimize the sum of squared error loss. In parallel to this machine learning approach, in [3,4], a 3D face model combined with specially developed algorithms was employed, in which depth information was captured by the camera for the head pose estimation task. 

Some deep learning-based approaches have also generated findings. For example, in [7], a CNN-based model was developed, in which the classification and regression were integrated to evaluate approximate regression confidence. Their results demonstrated that the training of the CNN can achieve near saturation with both 2D and 3D facial landmark-labeled datasets. In addition, in [3], a residual network was integrated with landmark localization structures. In [18], a Face-pose-Net network was built, showing how a simple CNN can be precisely trained and robustly regressed to head pose directly from a single image. In [4], to tackle the face alignment issue, an iterative approach for learning an effective Heatmap-CNN regressor was introduced for unrestrained face crucial points estimation and pose estimation.

Although a great deal of work exists in this area to enhance the accuracy of landmark detection, the reliance on landmark detection hinders its performance in the cases of a significant area occlusion and substantial angle deflection.

### 2.2. Estimation without Key Points

With the remarkable performance of deep learning approaches in different tasks in the CV field, head pose estimation models independent of landmarks are developed. In [14], a CNN paired with adaptive gradient algorithms was employed to achieve estimation under field datasets without depending on important points, but the estimation precision is unideal. Thereafter, a novel milestone of landmark-free head pose estimation was achieved in [15], which employed the fundamental Resnet-50 structure [20] and classified the head pose into an interval by 3°. In [10], the concept of soft stagewise regression was presented, and a fine-grained structural mapping of spatial features was employed to discover the spatial relationship between features. Shortly thereafter, in [3], a feature decoupling module was added into the CNN, which can explicitly learn the discriminative features of each bit pose by adaptively calibrating the channel response and bounding the variable subspace distribution.

In addition, by means of the angular annotation of the dataset, it is demonstrated that the labeling of quaternion or Euler angles can lead to discontinuities in angle estimation [31]. In order to solve the non-stationary problem (that is caused by labeling datasets using Euler angles), on the one hand, L2 loss was integrated with regression loss based on quaternion [16]; on the other hand, a rotation matrix was applied. For instance, in [30], the Frobenius norm’s solution was computed by replacing the singular value decomposition with fundamental algebraic operations. In [48], a two-dimensional Lorentz distribution and angular weight assignment were applied to solve the problems caused by uneven label distribution. In [49], an anisotropic angular distribution learning (AADL) network was proposed, in which Kullback–Leibler scatter was chosen to measure the predicted labels and the ground truth labels. In [12], the matrix Fisher distribution was presented, using the rotation matrix to model the head rotation uncertainty. In the latest study [11], the head pose was represented as three vectors and the model performance was evaluated using the mean absolute error of vectors (MAEV).

In summary, in the above methods, the features related to head pose were generally learned autonomously through neural networks, which did not require additional key point information and can return the head pose directly from the image perspective. Although the addition of the rotation matrix can efficiently eliminate the angle estimation discontinuity, the loss function or even the model itself needs to be further redesigned and improved.

### 2.3. Multitask and Feature Pyramid

Previously, several estimation tasks were conducted simultaneously using multitask estimation approaches under one CNN model. For example, in [28], CNNs with residual blocks and lateral skip connections were employed to simultaneously perform landmark-based face alignment and head pose estimation. Similarly, a cascaded structure was employed in [27] for face alignment and face detection tasks, which improved the performance significantly due to the fact that the correlation within tasks can contribute to facilitating the complementary information of each other. Similarly, this inter-task synergy was also specifically explained by [28]. In [25], model construction and selection related to multitask convolution were explained in detail. In 2021, a fine-feature encoder and three decoders were employed to achieve estimations for three different tasks [29].

At the same time, the idea of multi-scale prediction emerged in target detection. For example, the idea of feature pyramids was proposed in [40] to efficiently capture small-scale information that is usually neglected in deep layers. A global-and-local transformation was used in [44], aiming to solve the reconfiguration problem and reuse of feature hierarchies in the process of constructing feature pyramids. Recently, top-down and bottom-up feature connections were proposed in [41], integrating features at various scales. Furthermore, an adaptive spatial feature-fusion structure was proposed in [43], which can spatially filter conflicting information to delete inconsistency.

To the best of our knowledge, the estimation of three head pose angles has been considered as three branches belonging to the same task and sharing the same layers. However, this increases the burden of model tuning for each angle. Inspired by the multitasking output, in this study, the three angles of the head pose are considered as three different tasks, which are assigned to the three network layers and the corresponding feature scales are enriched using a feature pyramid.

## 3. Method

In this section, first, the basic process of head pose estimation is outlined, and then the proposed THESL-Net model is described in detail. Second, a concept of tiered estimation is proposed and the modified loss function is given.

### 3.1. Problem Formulation

Generally, the head pose estimation can be summarized by the following steps. Given a set of face images $X=\left\{x_{n}|n=1,\ldots ,N\right\}$ and the pose vector $y_{n}$ for each image $x_{n}$, where N represents the image number, the elements of $y_{n}$ comprise the angles of yaw, pitch, and roll, denoted as ϕ, θ, and ψ, respectively. The aim is to discover a mapping function F by minimizing the mean absolute error (MAE) with respect to the estimation $\hat{y}=F\left(x\right)$ and ground truth y:

(1) $$F\left(X\right)=\frac{1}{N}\;\sum_{i=1}^{N}\left|\left|{\hat{\phi }}_{i}-\phi_{i}\right|+\left|{\hat{\theta }}_{i}-\theta_{i}\right|+\left|{\hat{\psi }}_{i}-\psi_{i}\right|\right|$$

where ${\hat{\phi }}_{i}$, ${\hat{\theta }}_{i}$, and ${\hat{\psi }}_{i}$ represent the estimations of ${\hat{y}}_{i}$ after the target of evaluation is split into three different angles.

### 3.2. Overview of THESL-Net

**Figure 2:**
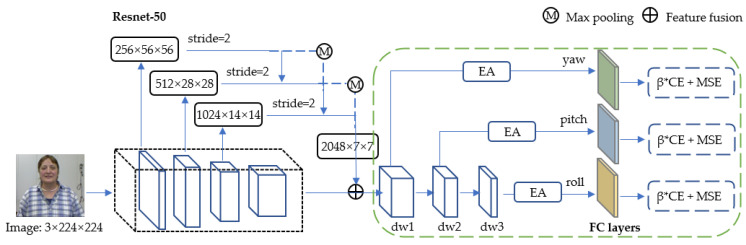
Structure and details of the proposed THESL-Net model, where EA represents external attention and dwi∣i=1, 2, 3 represents different downscaling layers.

The framework of the proposed THESL-Net model is shown in Figure 2. The proposed model comprises one backbone and one tiered estimation module. In particular, the proposed THESL-Net model is an end-to-end model, and the backbone is Resnet-50 with a feature pyramid structure. Ideally, the loss predicted by the proposed model should have a similar growth trend as that of the real loss; thus, a limiting factor β is added to the cross-entropy loss used in this study.

After the fixed-size images go through the model, a feature mapping is obtained at each stage of the backbone network, and the features extracted from neighboring stages are fused using down-sampling and maximum pooling to maintain c×w×h constant. The final fused features are input into the tiered estimation module, and three head branches with varied parameters are generated by minimizing the channel number. The traditional regression and classification loss are employed to compute the total estimation loss in the training process, where each head branch is spread out by a linear layer. Furthermore, external attention is used to perform feature selection [37], which better differentiates the three angles.

Details on feature fusion, tiered estimation, and limitations on the loss function will be depicted in the following subsections.

### 3.3. Tiered Estimation 

Three linear layers, each of which is responsible for predicting a single vector, are commonly employed in head pose estimation. The three linear layers share the same convolutional layer parameters, as shown in Equation (2):

(2) $$\left[\hat{\phi },\hat{\theta },\hat{\psi }\right]=\left[K_{1}\Gamma +b_{1},K_{2}\Gamma +b_{2},K_{3}\Gamma +b_{3}\right]$$

where K denotes the various weights, Γ denotes the feature obtained by the convolution layer, and b represents the bias factor. Suppose the estimation loss of an image is $L\left(\hat{y},y\right)=\left[0,5,10\right]$. Since the network layer is shared in gradient backpropagation, the estimation loss after tuning can be denoted as $L\left(\hat{y},y\right)=\left[2,3,5\right]$. Although the total predicted loss is lowered, it is not the best model for yaw.

Inspired by the idea of feature pyramid network, a tiered structure is developed in this study. In the feature fusion, only the down-sampling technique is adopted, and the estimation findings under various scales are not fused. For the 1/2 ratio case, a 3 × 3 convolution layer with a stride of 2 is employed; for the 1/4 ratio case, a two-step max-pooling layer is added before the 2-stride convolution; and for the 1/8 ratio case, fusion is not applied, as shown in Figure 2. Each phase of the backbone network is denoted by S, the features are fused as follows:

(3) $$S_{j}=\gamma_{1}S_{j}+\gamma_{2}S_{j-1}^{\to j}+\gamma_{3}S_{j-2}^{\to j}$$

where $S_{j}\;|\;j=3,\;4$ denotes the last two stages, →*j* denotes fusion with the current layer as the spatial scale standard, and γ represents the fusion weight. When j is equal to 1 or 2, $\gamma_{2}$ or $\gamma_{3}$ is 0, respectively. Similar to [43], we force $\gamma_{1}+\gamma_{2}+\gamma_{3}=1\;|\;\gamma_{1},\gamma_{2},\gamma_{3}\in \left[0,1\right]$. Particularly, three 1×1 convolution layers are employed to compute the weight scalar maps for each of $\lambda_{\gamma_{1}}$, $\lambda_{\gamma_{2}}$, and $\lambda_{\gamma_{3}}$ from $\gamma_{1}$, $\gamma_{2}$, and $\gamma_{3}$, respectively.

(4) $$\gamma_{1}=\frac{e^{\lambda_{\gamma_{1}}}}{e^{\lambda_{\gamma_{1}}}+e^{\lambda_{\gamma_{2}}}+e^{\lambda_{\gamma_{3}}}}$$

In the tiered estimation module, a 3 × 3 convolution layer with padding of 1 is employed to maintain the spatial resolution unchanged, as 1/2 spatial scale ratio downscaling is performed three times, generating features $dw_{1}$, $dw_{2}$, and ${dw}_{3}$ in sequence. The external attention comprises two layers of 1×1 convolution that are responsible for the common feature selection in the dataset. Then, softmax is conducted on the probability matrix of yaw, pitch, and roll, which are generated from the linear layer. From this, the interaction between the three angles is weakened, as shown in Equation (5):

(5) $$\left[\hat{\phi },\hat{\theta },\hat{\psi }\right]=\left[K_{1}\Gamma_{1}+b_{1},K_{2}\Gamma_{2}+b_{2},K_{3}\Gamma_{3}+b_{3}\right]$$

where $\Gamma_{1}$, $\Gamma_{2}$, and $\Gamma_{3}$ are the parameters of $dw_{1}$, $dw_{2}$, and $dw_{3}$, respectively. $\Gamma_{1}$, $\Gamma_{2}$, and $\Gamma_{3}$ are related to each other as follows:

(6) {Γ2=W1Γ1+b4  Γ3=W2Γ2+b5  

In Equation (6), $W_{1}$ and $W_{2}$ are parameters of the new convolution, and $b_{4}$ and $b_{5}$ are new bias terms.

**Figure 3:**
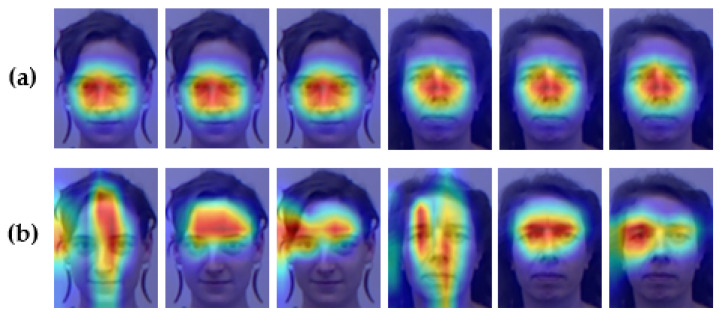
Heatmap of three angle branches of various models. From left to right: yaw, pitch, and roll. (**a**) Hope-Net and (**b**) the proposed THESL-Net.

In the proposed model, head pose estimation is considered to be three tasks, and additional tuning space is also employed. As demonstrated in Figure 3, Grad-CAM [50] is used to visualize the original single-branch structure and the proposed three-branch structure (i.e., dw1, dw2, and dw3), aiming to show the changes brought about by the tiering: the areas of concern are no longer identical between the three angles.

### 3.4. Dynamic Loss Adjustment

Rotation matrices are employed to solve the angle discontinuity challenges caused by a quaternion or Eulerian angle labeling, although effective, specially designed models are often required. However, it is discovered that the loss function’s incoherence is another cause of the discontinuity; in detail, this discontinuity in angle estimation is due to the classification loss being larger than the MSE loss at about 1° from the classification edge. Taking a single picture as an example, the typical loss function is as follows:

(7) $$L\left(\hat{y},\;y\right)=-\overset{k}{\underset{i=1}{\sum }}Y_{ic}\log\left(\sigma ({\hat{Y}}_{i})\right)|L_{ce}+L_{mse}$$

where k represents the number of categories; $Y_{ic}$ is 0 or 1, corresponding to whether the classification is correct; ${\hat{Y}}_{i}\;$ is the probability matrix; and σ denotes softmax.

Another simple example to illustrate the loss imbalance at both ends of the classification is as follows. Set the ground truth to [0°, 3°, 5°] and estimation to [1°, 3.5°, 7°], and then divide (−99°, 99°) into 66 groups with 3° as an interval. When the truth error between estimation and ground truth is within 1°, the regression task appears in two cases: the estimation is correctly classified, which is called intra-class regression, or the estimation is incorrectly classified, which is called inter-class regression. To be specific, in the case when the estimation is intra-class, the cross-entropy loss is minimal, and the total loss follows the truth loss trend. However, in the case when estimation is inter-class, the cross-entropy loss is larger than the mean squared loss (because of the index of 2), leading to the total loss being inverse to the truth loss trend, as stated in Section 1. This makes the model difficult to learn.

In [15], a coefficient α=2 is provided for the MSE, as shown in Equation (8):

(8) $$L\left(\hat{y},y\right)=L_{ce}+\alpha {\left(\sum_{i=1}^{k}dY_{id}\sigma ({\hat{Y}}_{i})-99-y\right)}^{2}\;|\;L_{mse}$$

where *d* represents the category length and $Y_{id}$ represents the category label. Here, 99 is the regression constant term, as a result of restricting the angle to between −99° and 99° during the processing of the dataset. When $L_{mse}$ is considerably small, multiplying by a factor α=2 can relatively alleviate the discontinuity problem caused by the loss function. However, it does not capture the matter’s crux and can further increase this incongruity when an intra-class loss is greater than an inter-class loss.

Considering the synergy present between the two losses, we set an additional constraint for classification loss:$\;\beta ={\left(\hat{y}-y\right)}^{2}/({\left(\hat{y}-y\right)}^{2}+1$). Then, the cross-entropy loss after the update is given by

(9) $$L_{ce^{\prime }}=-\frac{{\left(\hat{y}-y\right)}^{2}}{(\left(\hat{y}-y{)}^{2}+1\right)}\overset{k}{\underset{i=1}{\sum }}Y_{ic}\log\left(\sigma \left({\hat{Y}}_{i}\right)\right)$$

After restriction, β∈[0,1] is also added to the backpropagation gradient, making the resulting penalty small when the true loss is small. In the above example, when multiplying by β, the CE loss of pitch can be lowered to 1/5 of its original. This resets the model’s total loss to the same trend as the true loss. Subsequently, 2β is employed to improve the error penalty for loss above 1°, which can accelerate the model’s convergence.

To confirm the effectiveness of the proposed approach, in our study, another set of loss functions is developed based on the rotation matrix, as demonstrated in Equation (10), which comprises the MSE and MAEV. The concept is that the vectors corresponding to the three angles in the rotation matrix must be perpendicular to each other, or else a penalty is given.

(10) $$L\left(\hat{y},\;y\right)=L_{mse}+\frac{1}{3}\mu \underset{i\neq j}{\sum }V_{t}^{\left(i\right)}V_{p}^{\left(j\right)}\;|\;L_{maev}\;\;$$

where $V_{t}$ represents the rotation matrix of ground truth; $V_{p}$ represents the estimation rotation matrix; i,j denote vectors of yaw, pitch, roll, i,j=1,2,3; and μ denotes the range [0.1, 0.5] given by [11]. The experimental findings reveal that our loss-limiting approach (i) has similar performance to the rotation matrix-based approach under the same conditions and (ii) can solve the discontinuity problem from two aspects, as demonstrated in Section 4. Algorithm 1 details the proposed approach’s training process.
**Algorithm 1** Training procedure for the Tiered estimation network with self-adjust loss**Input:** A batch of images *T* and the hyper parameters *β*.**Output:** The loss reverse gradient ▽*l*ϕ, ▽*l*θ, ▽*l*ψ.1: Initialize the reverse gradient ▽*l*ϕ, ▽*l*θ, ▽*l*ψ;2: **for**
*t* = 1,…, *T* **do**3:      Extracting the feature matrix Y =(Yϕ, Yθ, Yψ);4:      Selecting features by EA $Y^{e}$$=(Y\phi^{e}$$,\;Y\theta^{e}$$,\;Y\psi^{e}$);5:      Computing by tiered $Y^{t}$ $=(Y\phi^{t1}$, *Y*$\theta^{t2}$, *Y*$\psi^{t3}$) with (5);6:      Calculating loss L=(Lϕ, Lθ, Lψ) with (7);7:      Limiting cross-entropy loss $L_{ce}$ $=\beta L_{ce}\;$ with (9);8:      ▽*l*ϕ, ▽*l*θ, ▽*l*ψ ← (▽*l*ϕ, ▽*l*θ, ▽*l*ψ) *β*;9: **return** ▽*l*ϕ, ▽*l*θ, ▽*l*ψ.

### 3.5. Optimization

**Figure 4:**
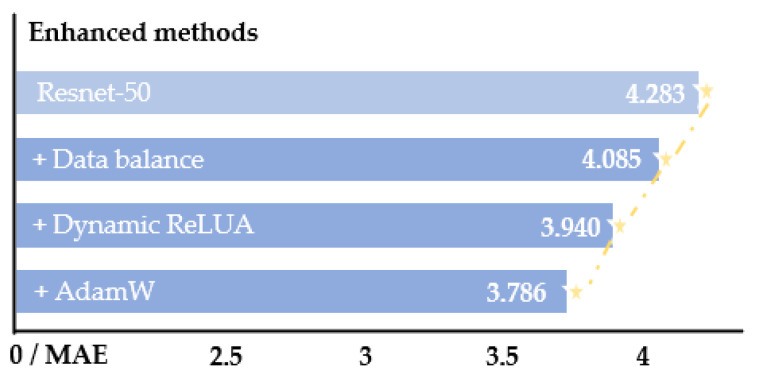
Model improvement on the BIWI dataset. In total, 70% of data is employed for training, and the remaining is for testing.

To further improve the proposed model, a series of measures are employed to enhance the baseline of Resnet-50 as the backbone, and the enhancements caused are also listed, as shown in Figure 4. First, the dataset is kept in balance using both oversampling and left–right mirroring with Hopenet [15] as the benchmark. The distribution ratio of the large, medium, and small (about 30° for each interval size) angles is 2:2:1 in the balanced dataset. Then, according to the previous research on the Resnet network and transformer structure [35,36] the ReLU is modified to the Dynamic ReLU stated in [47] to enhance the model’s representation ability, and the AdamW optimizer [46] instead of the Adam optimizer is employed to enhance the model’s generalization. The combination of these approaches leads to a 0.5° reduction in baseline loss.

## 4. Experiments

### 4.1. Implementation Details

Pytorch is used to implement the proposed network. All images are cropped to 224×224 size (surrounding the face) and then normalized using transform mean and standard deviation. During training, random masks are introduced to all images using CutOut. An AdamW optimizer with a weight decay of $1\times 10^{-5}$ is employed, the learning rate is set to $\;1\times 10^{-3}$, the learning rate decayed is set to the original 0.9 every 20 epochs, and the loss-limit factor is set to 2β. In addition, the linear layer’s learning rate is adjusted to $5\times 10^{-3}$, and both the first convolution layer and batch norm layer are kept frozen. The model is trained for 200 epochs with a batch size of 64, and four GTX 1080Ti GPUs are employed for this process.

### 4.2. Datasets and Evaluation

**Figure 5:**
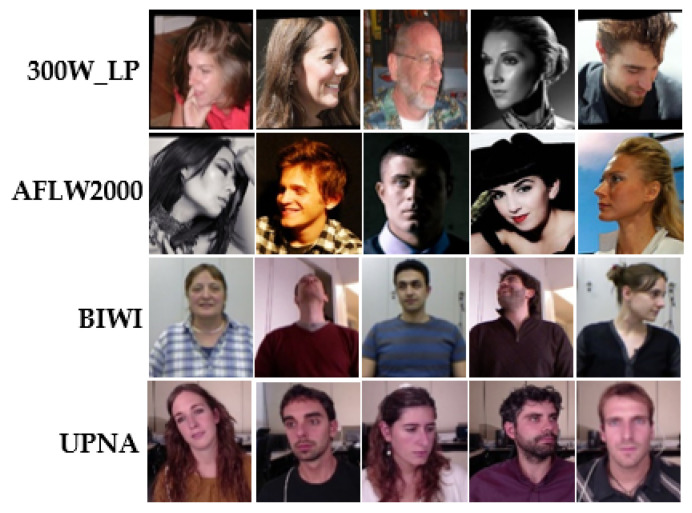
Some samples of datasets, from top to bottom are 300W-LP, AFLW2000, BIWI, and UPNA.

As shown in Figure 5, the proposed model is examined on four popular public benchmark datasets: 300W-LP [21], BIWI [23], AFLW2000 [22], and UPNA [24].

**300W-LP**: The 300W-LP [21] dataset is an extended version of the 300 W [51] dataset, which has over 120 k images for face alignment with 68 landmarks.

**BIWI**: The BIWI dataset [23] has 24 videos produced from 20 subjects, totaling 15,678 frames, each corresponding to both RGB and depth images. Since face position is not offered in this dataset, in our study, Yolo5-face [52] is employed to produce the persons’ head borders.

**AFLW2000**: The AFLW2000 [22] dataset is derived from the first 2000 images in the AFW [53] dataset with 68 landmarks. Faces in this dataset have complicated pose variations and backgrounds.

**UPNA**: The UPNA [24] dataset has 10 groups, each with 12 videos from one subject. Each video contains only a single direction of head pose variation and uses 54 landmarks, totaling 36,000 images. The face deflection range in this dataset is small and solitary.

For comparison with other the-state-of-the-art approaches, as stated in Hopenet [15], FSA-Net [10], and TriNet [11], the same training and testing setup is used in our study, and the images with Euler angle deflection outside of −99° to 99° are filtered out. In particular, it is discovered that the angle distributions of the UPNA and BIWI datasets are between [−48°, 36°] and [−75°, 85°], respectively. Figure 5 shows samples of the datasets, and this study is conducted in the following two scenarios:

(1) The model is trained and evaluated on the datasets of 300W-LP, BIWI, AFLW2000, and UPNA.
(2) In total, 70% of the BIWI and UPNA datasets are employed for training and 30% for testing. The train set is not crossed with the test set. For example, in the BIWI dataset, 16 videos are employed for training and 8 videos for testing.

In all of the above studies, to assess the performance of the proposed model, the MAE is used as the loss function.

### 4.3. Competing Methods

To show the effectiveness, we compare the proposed approach with other state-of-the-art approaches on public benchmark datasets, with data from either the original article or experimental findings.

The following is a brief description of previous work related to the proposed model, all based on RGB images. Dlib [1] addresses 2D to 3D fitting challenges by matching face landmark points for head pose estimation. 3DDFA [21] employed a CNN to develop an approach for fitting 3D face models to 2D images that skips the step of facial landmark detection. There are also more popular methods that do not rely on key points. For example, Hopenet [15] suggested a concept of head pose estimation without key points based on Resnet-50, considerably enhancing the model’s performance under complex scenes. Thereafter, FSA-Net [10] introduced the idea of soft stagewise regression and developed a fine-grained structural mapping to capture spatial features. QuatNet [16] employed a multivariate loss function based on quaternion to address the difficulty of the non-stationary property caused by Euler angle representation. FDN [13] elaborates a feature decoupling network with cross-category center loss to restrict the distribution of the latent variable subspaces. MFDNet [12] constructed the triplet module and the matrix’s Fisher distribution module to address the uncertainty of head rotation. TriNet [11] re-labeled dataset samples using orthogonal constraints on the three vectors and assessed them using MAEV. To enhance the accuracy of head pose estimation for drivers, ref. [54] proposed a spatial temporal vision transformer (ST-ViT) model, taking a pair of image frames rather than one single frame as the input.

### 4.4. Experiment Results

We explore the performance variation of the model using different backbone networks. The comparison between three various backbones (including ResNet-50, ResNext-101, and the latest ConvNext) is given in Table 1. Notably, in this study, all orientations are shown in degrees.

First, we note that “w” denotes with the proposed method (see the odd rows in Table 1), and “w/o” denotes without the proposed method (see the even rows in Table 1). The comparison between the odd and even rows shows that the proposed method can improve the model performance for all three backbones. Taking ResNet-50 as an example, by introducing the proposed method, the average MAE value (of yaw, pitch, and roll) on the AFLW 2000 dataset can be improved from 6.16° to 4.40°, and the average MAE value (of yaw, pitch, and roll) on the BIWI 2000 dataset can be improved from 5.18° to 3.56°.

Second, the comparison between the three backbones show that the best performance can be achieved by using ResNet-50. Taking the validation on the AFLW 20,000 dataset, for example, the MAE values on ResNet-50, ResNet101, and ConvNext are 4.40°, 5.62°, and 7.84°, respectively. Since the best results are achieved with the ResNet-50 backbone, the experiments will be conducted on ResNet-50.

Table 2 and Table 3 show the findings of our proposed model, which is compared with other state-of-the-art approaches. We note that the proposed model is trained on the 300W-LP dataset. In Table 2, the test results on the AFLW2000 dataset are shown. From this table, we can see that the proposed model THESL-Net attains the minimum error on a roll, and the MAE is somewhat higher than that of MFDNet, but the structure of the proposed approach is much simpler and thus can be readily conducted on other models. Furthermore, in Table 3, the test results on the BIWI dataset are shown. From this table, we can see that THESL-Net realizes the best performance with an MAE reduction of 0.06° compared to the second-best approach (MFDNet). The proposed approach does not rely on landmark detection, and the loss limitation factors can be adjusted automatically with the evaluation process without additional settings.

Table 4 reveals the findings compared with other approaches on the BIWI dataset, where 70% and 30% of the data were employed for training and testing, respectively, without crossover. All compared methods are based on RGB, and the finding of Hopenet [15] are derived from re-runs in [11]. THESL-Net is first fine-tuned, resulting in the best finding on yaw, and the MAE decreases by 0.36° compared to the second place. Other indicators are also in the upper middle position, which indicates the effectiveness of our tiered estimation concept.

The performance of the proposed method on the UPNA dataset is given in Table 5, where ‘/’ means the corresponding value is not given in the original article. To make a fair comparison, we make up the experiment by using 90% of the UPNA dataset for training and 10% of the UPNA dataset for testing. From this table, it can be seen that the best MAE was achieved by the proposed method when using the same dataset-partitioning method. 

**Figure 6:**
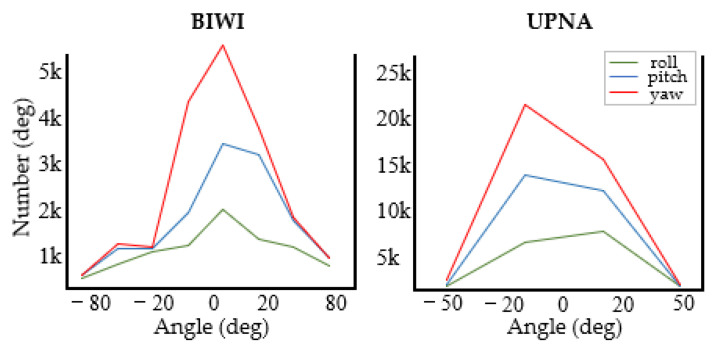
Comparison of angle range [−48°, 36°] in the UPNA dataset and angle range [−75°, 85°] in the BIWI dataset.

To examine the influence of head deflection angle range on the proposed model, we further compare the BIWI dataset with the UPNA dataset and generate the findings as shown in Figure 6. Both datasets are obtained in an experimental setting with low disturbance, containing three angles of different intervals. We only employ the MAE to evaluate the change in model performance. The experimental findings reveal that the proposed model has good performance in various angle ranges. Table 6 shows the details. Equation (10) shows further development of a new loss function, which consists of MSE and MAEV. It is compared with the proposed approach to show the extent to which the loss function and labeling affect the angle estimation discontinuity, as shown in Table 6.

By combining the loss limitation and the rotation matrix, as shown in Equation (11), the overall loss increases instead.

(11) $$L\left(\hat{y},y\right)=2\beta L_{ce}+L_{mse}+L_{maev}$$

A reasonable explanation is that loss-limiting and labeling approaches have similar influences, and simply adding them together equals 4β, which destroys the loss function’s coordination within 1° of the prediction error again.

### 4.5. Visualization

**Figure 7:**
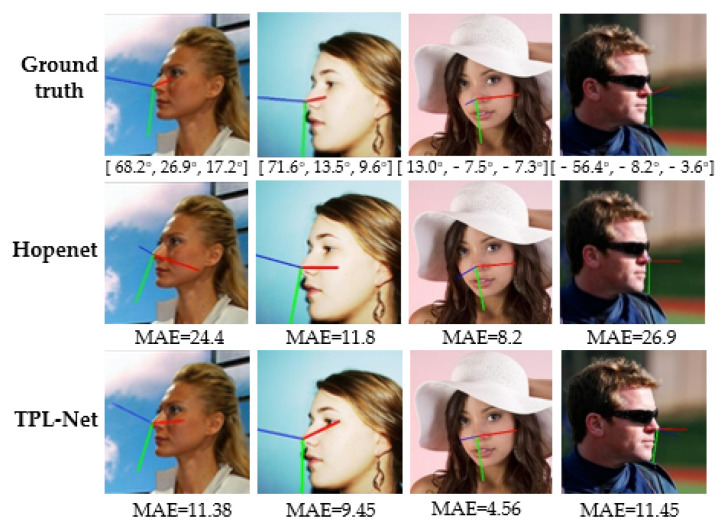
Comparison of head pose estimation in the AFLW2000 dataset for significant angle deflection and masking cases.

In this section, the process of model training and the comparison between different approaches are visualized. First, Figure 7 shows the performance of the proposed approach in the case of occlusion and significant angle deflection. We have selected a part of the images with significant angle deflection in the AFLW2000 dataset. Both the Hopenet and THESL-Net models, which have a similar backbone, are employed to forecast the head pose. We plot various colored lines to visualize the head deflection, where the blue, green, and red lines are used to indicate the front, bottom, and side of the face, respectively. Our approach minimizes the MAE by more than 10° in deflection cases and also reduces the MAE by about 4° for the case where the face is obscured.

**Figure 8:**
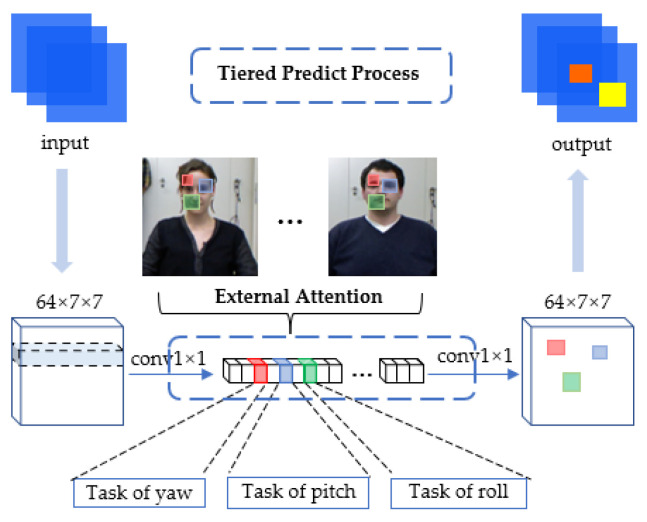
Visualization of the tiered estimation process (showing the estimated task of yaw, pitch, and roll in red, blue, and green colors, respectively).

Figure 8 shows the function of the tiered estimation module in the training process. A batch of features generated from the backbone network is taken as input, and then a 1 × 1 convolution layer is used to deflate the number of channels. The three colors in the figure denote the respective regions of interest in the estimation task of yaw, pitch, and roll. Finally, the features after weight assignment go through a layer of 1×1 convolution to reduction channel numbers before outputting to the linear layer. Notably, we use the external attention mechanism to detect common features among different character samples, although other tasks may require different attention mechanisms. The concept of tiered estimation minimizes the influence of fine-tuning between the three angles.

**Figure 9:**
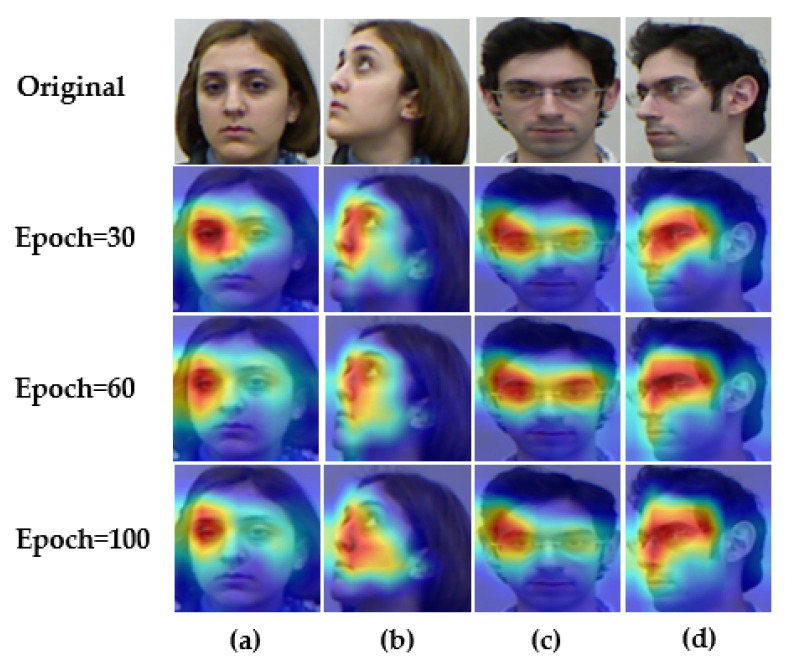
Visualization of the model regions of interest in training. A total of 100 epochs were trained on the BIWI dataset. (**a**–**d**) are people in the same posture but with different identities.

Furthermore, demonstrating the changes more explicitly in the model during the training process, Grad-CAM [50] is employed to visualize the areas that the model focuses on before the tiered layer, as shown in Figure 9: columns (a) and (c) have separate identities, columns (a) and (b) have different postures, and columns (a) and (d) are both different. As the training epochs improve, the external attention makes the model’s area of interest gradually focus on those common features, which leads to good robustness in the head pose estimation model for people with a similar pose, but separate identities. Additionally, for the same person, the regions that the model focuses on are alsso different for different head poses. This indicates that the proposed model is simultaneously identity-robust and pose-robust.

### 4.6. Ablation Study

In this section, the effect of different blocks (tiered estimation module and various loss limits) on the THESL-Net model performance is investigated. The ablation studies are performed following the enhancement of Resnet-50; the three techniques used are shown in Figure 4. For this, two sets of studies are developed. The first set is trained on 300W-LP and tested on the AFLW2000 and BIWI datasets. The second set employs 70% of each of the BIWI and UPNA datasets as the training set, and 30% as the test set. Each set of studies examines the influence of with/without the tiered idea and with β/2β/without loss limit on the findings differently. The experimental findings are shown in Table 7 and Table 8.

As observed in Table 7, the MAE of the base model is 5.65° on AFLW2000 and 4.82° on the BIWI dataset when either module is not used. However, the model performance is significantly improved when either of the two modules is added alone. Among them, the performance of THESL-Net is optimal when using the tiered module with loss limit =2β, which reduces by 1.25° and 1.26° on the AFLW2000 and BIWI datasets, respectively. This shows that both of our strategies are effective.

**Figure 10:**
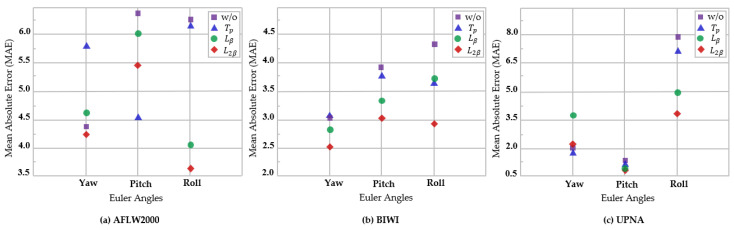
Comparison of the MAE of various components at various angles under AFLW2000, BIWI, and UPNA. THESL-Net is divided into with or without tiered module, and with or without different loss limits.

In Table 8, we introduce the ablation findings for the BIWI and UPNA datasets, which have different angle distribution ranges, whereas the UPNA dataset alone has a smaller and more concentrated one. The losses using the best combination in the BIWI and UPNA datasets are minimized by 0.94° and 1.39°, respectively, and the final MAE of the two are not considerably different, indicating that our model performs well at various angle ranges. Figure 10 further reveals the details of the experimental findings for each module of the model at various angles. As seen from the figure, using the combination of the two techniques always attains optimal findings.

## 5. Conclusions

To overcome the two challenges in the field of head pose estimation, in this study, THESL-Net is proposed, which comprises the tiered estimation module and the loss-limit component. To be specific, to solve the problem of mutual interference between the angles in regulation, the tiered structure forms three branches by dimensionality reduction, corresponding to the three angles of the head pose estimation. By separating the three angles’ network parameters, the mutual interference between the yaw, pitch, and roll tuning is substantially decreased, which makes the estimation loss have space for more reduction. In addition, to solve the problem of discontinuity in angle prediction, unlike the rotation matrix-based approach, we solve the problem from the perspective of the loss function by restricting the loss function, while the effect is comparable to that of the rotation matrix. 

On the popular public standard datasets AFLW2000, BIWI, and UPNA, the experimental findings reveal that our approach has better identity robustness than previous approaches and demonstrates state-of-the-art performance.

## Figures and Tables

**Table 1:** Performance comparison between various backbones.

Backbone	With/Without	AFLW2000				BIWI			
		Yaw	Pitch	Roll	MAE	Yaw	Pitch	Roll	MAE
ResNet-50	w/	4.22	5.42	3.56	**4.40**	3.53	4.64	2.51	**3.56**
ResNet-50	w/o	6.47	6.56	5.44	6.16	5.17	6.98	3.39	5.18
ResNet101	w/	6.63	5.19	6.02	**5.62**	4.43	5.13	2.91	**4.15**
ResNet101	w/o	6.70	7.89	4.33	6.31	2.63	4.98	6.18	4.60
ConvNext	w	12.06	5.27	6.20	**7.84**	3.90	7.87	6.83	**6.19**
ConvNext	w/o	14.12	8.18	6.74	9.68	6.82	6.30	9.46	7.53

**Table 2:** Comparisons on the AFLW2000 dataset (all methods are trained on the 300W-LP dataset).

Method	Yaw	Pitch	Roll	MAE
Dlib [1]	23.1	13.6	10.5	15.8
3DDFA [21]	5.40	8.53	8.25	7.39
Hopenet [15]	6.47	6.56	5.44	6.16
FSA-Net [10]	4.50	6.08	4.64	5.07
QuatNet [16]	3.97	5.62	3.92	4.50
FDN [13]	**3.78**	5.61	3.88	4.42
MFDNet [12]	4.30	**5.16**	3.69	**4.38**
TriNet [11]	4.04	5.77	4.20	4.67
**THESL-Net**	4.22	5.42	**3.56**	4.40

**Table 3:** Comparisons on the BIWI dataset (all approaches are trained on the 300W-LP dataset).

Method	Yaw	Pitch	Roll	MAE
Dlib [1]	16.8	13.8	6.19	12.2
3DDFA [21]	36.2	12.3	8.78	19.1
Hopenet [15]	5.17	6.98	3.39	5.18
FSA-Net [10]	4.27	4.96	2.76	4.00
QuatNet [16]	4.01	5.49	2.94	4.15
FDN [13]	4.52	4.70	2.56	3.93
MFDNet [12]	**3.40**	4.68	2.77	3.62
TriNet [11]	4.11	4.76	3.05	3.97
**THESL-Net**	3.53	**4.64**	**2.51**	**3.56**

**Table 4:** Comparisons of the BIWI dataset (70% of the BIWI dataset is employed for training and 30% for testing).

Method	Yaw	Pitch	Roll	MAE
Hopenet [15]	4.33	4.42	4.09	4.28
FSA-Net [10]	2.89	4.29	3.60	3.60
FDN [13]	3.00	3.98	2.88	3.29
MFDNet [12]	2.99	3.68	2.99	3.22
TriNet [11]	2.93	3.04	**2.44**	**2.80**
ST-ViT [52]	3.27	**2.82**	3.12	3.07
**THESL-Net**	**2.53**	3.08	2.95	2.85

**Table 5:** Comparisons of the UPNA dataset (90% of the UPNA dataset is employed for training and 10% for testing).

Method	Yaw	Pitch	Roll	MAE
Nao [55]	/	4.10	2.50	3.30
ResNet50+YL2 [56]	2.49	3.89	/	3.19
Dense 3D [57]	0.98	2.71	1.53	1.74
AAM+POSIT [24]	1.04	1.63	2.19	1.62
**THESL-Net**	1.32	2.05	**1.16**	**1.54**

**Table 6:** Comparison with the rotation matrix-based approach on the BIWI dataset with 70% of the dataset for training and 30% for testing.

Loss	Yaw	Pitch	Roll	MAE
Ours (Equation (9))	2.53	3.08	2.95	**2.85**
MSE + MAEV	2.67	3.19	2.88	2.91
Ours + MAEV	2.59	3.73	2.85	3.06

**Table 7:** Ablation study over different components (with/without tiered module and with/without loss limit) on the AFLW2000 and BIWI datasets. All methods are trained on the 300W-LP dataset.

Tiered Module	Loss-Limit	AFLW2000				BIWI			
		Yaw	Pitch	Roll	MAE	Yaw	Pitch	Roll	MAE
w/o	w/o	4.37	6.33	6.24	5.65	7.14	4.38	2.94	4.82
w/	w/o	5.75	4.50	6.18	5.48	3.05	5.87	4.28	4.40
w/o	β	3.53	4.06	7.49	5.03	4.83	3.63	5.21	4.56
w/	β	4.60	5.97	4.08	4.88	3.33	4.56	4.31	4.07
w/o	2β	4.38	5.06	4.71	4.72	3.16	3.94	4.30	3.80
w/	2β	4.22	5.42	3.56	**4.40**	3.53	4.64	2.51	**3.56**

**Table 8:** Ablation study over different components (with/without tiered module and with/without loss limit) on the BIWI and UPNA datasets. In total, 70% and 30% of the datasets were used for training and testing, respectively.

Tiered Module	Loss Limit	BIWI				UPNA			
		Yaw	Pitch	Roll	MAE	Yaw	Pitch	Roll	MAE
w/o	w/o	3.16	3.94	4.27	3.79	2.04	1.44	7.93	3.81
w/	w/o	3.17	3.75	3.60	3.51	1.91	1.30	7.14	3.45
w/o	β	2.77	4.34	3.47	3.53	3.62	1.14	5.40	3.39
w/	β	2.87	3.39	3.70	3.32	3.74	1.11	4.98	3.28
w/o	2β	3.05	3.31	3.30	3.22	1.93	1.21	4.65	2.60
w/	2β	2.53	3.08	2.95	**2.85**	2.35	1.12	3.79	**2.42**

## Data Availability

The four open-access datasets, AFLW2000, 300W-LP, BIWI, and UPNA, are used in our study. Their links are as follows: AFLW2000: http://www.cbsr.ia.ac.cn/users/xiangyuzhu/projects/3DDFA/main.htm; 300W-LP: http://www.cbsr.ia.ac.cn/users/xiangyuzhu/projects/3DDFA/main.htm; BIWI: https://www.kaggle.com/datasets/kmader/biwi-kinect-head-pose-database; UPNA: https://www.unavarra.es/gi4e/databases/hpdb (all of the above datasets accessed on 23 May 2022).
